# Host and Pathway Engineering for Enhanced Lycopene Biosynthesis in *Yarrowia lipolytica*

**DOI:** 10.3389/fmicb.2017.02233

**Published:** 2017-11-20

**Authors:** Cory Schwartz, Keith Frogue, Joshua Misa, Ian Wheeldon

**Affiliations:** Department of Chemical and Environmental Engineering, University of California, Riverside, Riverside, CA, United States

**Keywords:** carotenoids, HMG1, lipid metabolism, metabolic engineering, mevalonate pathway, synthetic biology

## Abstract

Carotenoids are a class of molecules with commercial value as food and feed additives with nutraceutical properties. Shifting carotenoid synthesis from petrochemical-based precursors to bioproduction from sugars and other biorenewable carbon sources promises to improve process sustainability and economics. In this work, we engineered the oleaginous yeast *Yarrowia lipolytica* to produce the carotenoid lycopene. To enhance lycopene production, we tested a series of strategies to modify host cell physiology and metabolism, the most successful of which were mevalonate pathway overexpression and alleviating auxotrophies previously engineered into the PO1f strain of *Y. lipolytica*. The beneficial engineering strategies were combined into a single strain, which was then cultured in a 1-L bioreactor to produce 21.1 mg/g DCW. The optimized strain overexpressed a total of eight genes including two copies of HMG1, two copies of CrtI, and single copies of MVD1, EGR8, CrtB, and CrtE. Recovering leucine and uracil biosynthetic capacity also produced significant enhancement in lycopene titer. The successful engineering strategies characterized in this work represent a significant increase in understanding carotenoid biosynthesis in *Y. lipolytica*, not only increasing lycopene titer but also informing future studies on carotenoid biosynthesis.

## Introduction

Carotenoids are an important and diverse class of aliphatic C_40_ molecules with a variety of applications in nutrition and human health. Due to their high level of conjugated double bonds, carotenoids absorb light, and most of their natural biological functions, such as capturing light for photosynthesis, take advantage of this characteristic (Armstrong and Hearst, 1996). Carotenoids also have beneficial properties for human health as antioxidants and neutraceuticals, and can be used as a food coloring agent (Ye and Bhatia, 2012). Lycopene, a relatively simple compound and the starting point for the synthesis of most other carotenoids, has antitumor properties and has been shown to be beneficial for coronary health (Vilchez et al., 2011).

Carotenoids, such as lycopene, are natively produced by a range of organisms from higher plants to bacteria (Armstrong and Hearst, 1996). Most carotenoids used for human applications are either harvested from these natural producers or are chemically synthesized from petrochemicals, and so metabolic engineering for the production of these molecules represents a valuable opportunity to lower production costs and environmental impacts (Ye and Bhatia, 2012). A range of different carotenoids, including lycopene, β-carotene, astaxanthin, and zeaxanthin, have been produced in the model metabolic engineering hosts *Escherichia coli* and *Saccharomyces cerevisiae*(Yamano et al., 1994; Albrecht et al., 1999; Alper et al., 2005; Zhou et al., 2015; Chen et al., 2016). A common solution identified in these works was engineering the methylerythritol-4-phosphate (MEP) and mevalonate (MEV) pathways in *E. coli* and yeast, respectively, to increase carotenoid precursor pools and flux to the desire compound.

An alternative strategy is to identify non-convention or non-traditional microbial hosts with native phenotypes and metabolisms biased toward high yield, high rate biosynthesis of the desire product (Liu et al., 2013; Gao M.R. et al., 2017; Löbs et al., 2017a,b). The yeast *Yarrowia lipolytica* is emblematic of this trend, as it has been the focus of a range of metabolic engineering studies due to its oleaginous nature and ability to grow on diverse substrates (Beopoulos et al., 2009; Zhu and Jackson, 2015; Hussain et al., 2016b). Many studies have focused on engineering high lipid production and accumulation (Blazeck et al., 2014; Rakicka et al., 2015; Qiao et al., 2017), and a range of genetic engineering tools have been developed to facilitate these efforts (Fickers et al., 2003; Gao et al., 2016; Hussain et al., 2016a; Schwartz et al., 2016, 2017a). Recently, *Y. lipolytica* has emerged as an attractive host for carotenoid production due to its ability to produce high levels of acetyl-CoA, a precursor for both lipids and carotenoids (Liu et al., 2013), and its ability to produce large lipid droplets, which sequester carotenoids and prevent membrane destabilization (Sung et al., 2007). A previous study engineered lycopene synthesis in *Y. lipolytica*, with controlled bioreactor studies producing upward of 16 mg lycopene/g dry cell weight (DCW) (Matthaus et al., 2014). Other carotenoids have also been produced, with one study producing 49 mg β-carotene/g DCW after extensive pathway engineering and media composition optimization (Gao S.L. et al., 2017).

**FIGURE 1:**
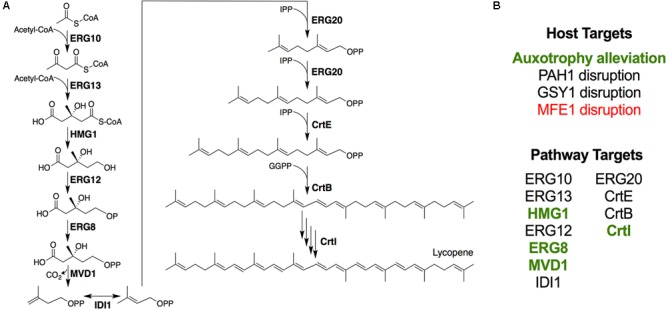
Engineering lycopene biosynthesis in *Y. lipolytica*. **(A)** Schematic of the lycopene biosynthetic pathway from acetyl-CoA. For simplicity, cofactors and ATP consumption is not shown. **(B)** Summary of strategies attempted in this work. Items shown in green enhanced lycopene yield, while items shown in red reduced lycopene yield.

In this study, we used advanced genetic engineering tools to, (1) characterize a series of gene disruptions that probed the effects of altering host cell physiology and metabolism, and (2) engineer enhanced flux down the mevalonate pathway to maximize lycopene production (**Figure 1**). We first characterized the effects of alleviating essential nutrient auxotrophies previously engineered into the PO1f strain of *Y. lipolytica*. Secondly, we characterized the effects of disrupting β-oxidation and glycogen biosynthesis. The effects of promoting membrane protein expression via disrupting PAH1, a mutation that putatively increases endoplasmic reticulum (ER) membrane abundance were also explored (Guerfal et al., 2013). In addition to exploring these host engineering strategies, we undertook a comprehensive set of enzyme overexpressions to increase lycopene production. Finally, the successful metabolic engineering strategies were combined to create a high producing strain that was characterized in fed-batch cultures.

## Materials and Methods

### Strains and Media

Yeast strains and plasmids used in this study are shown in **Table 1**. A derivative of the PO1f strain of *Y. lipolytica* (*MatA, leu2-270, ura3-302, xpr2-322, axp-2*; ATCC no. MYA-2613) (Madzak et al., 2000) that we previously described was used as the base strain for all genome editing and expression experiments in this study (Schwartz et al., 2017b). DH5α *E. coli* was used for plasmid construction and propagation, with growth in Lysogeny Broth (LB) supplemented with 100 mg/L ampicillin. Yeast cultures were grown in YPD medium (1% Bacto yeast extract, 2% Bacto peptone, 2% glucose), while cultures for lycopene biosynthesis were grown in YPD10 medium (1% Bacto yeast extract, 2% Bacto peptone, 10% glucose). For strain development, solid synthetic defined media without leucine and uracil [SD -Leu -Ura; 0.67% Difco yeast nitrogen base without amino acids, 0.067% CSM-Leu-Ura (Sunrise Science, San Diego, CA, United States), 2% glucose, 2% agar] was used to plate and screen transformants.

**Table 1:** Plasmids and strains used in this study.

	Description	Reference
**Plasmids**
pIW209	LEU2 pUAS1B8-TEF(136)-hrGFP	Blazeck et al., 2011
pIW245	URA3 pSL16-UAS1B8-TEF(136)-hrGFP	Schwartz et al., 2017b
pIW209-ERG20	UAS1B8-TEF(136)-ERG20-Cyc	This study
pIW209-MVD1	UAS1B8-TEF(136)-MVD1-Cyc	This study
pIW209-IDI1	UAS1B8-TEF(136)-IDI1-Cyc	This study
pIW209-ERG10	UAS1B8-TEF(136)-ERG10-Cyc	This study
pIW209-ERG13	UAS1B8-TEF(136)-ERG13-Cyc	This study
pIW209-ERG8	UAS1B8-TEF(136)-ERG8-Cyc	This study
pIW209-ERG12	UAS1B8-TEF(136)-ERG12-Cyc	This study
pIW209-HMG1	UAS1B8-TEF(136)-HMG1-Cyc	This study
pIW209-CrtE	UAS1B8-TEF(136)-CrtE-Cyc	This study
pIW209-CrtB	UAS1B8-TEF(136)-CrtB-Cyc	This study
pIW209-CrtI	UAS1B8-TEF(136)-CrtI-Cyc	This study
pIW245-CrtI	UAS1B8-TEF(136)-CrtI-Cyc	This study
pHR_XDH_hrGFP	1kb_XDH_up-UAS1B8-TEF-HMG1-CYC-1kb_XDH_down	Schwartz et al., 2017b
pHR_LEU2_hrGFP	1kb_LEU2_up-UAS1B8-TEF-MVD1-CYC-1kb_LEU2_down	Schwartz et al., 2017b
pHR_XDH_HMG1	1kb_XDH_up-UAS1B8-TEF-HMG1-CYC-1kb_XDH_down	This study
pHR_LEU2_MVD1	1kb_LEU2_up-UAS1B8-TEF-MVD1-CYC-1kb_LEU2_down	This study
pCRISPRyl_XDH	pCRISPRyl with XDH targeting sgRNA	Schwartz et al., 2017b
pCRISPRyl_LEU2	pCRISPRyl with LEU2 targeting sgRNA	Schwartz et al., 2017b
pCRISPRyl_MFE1	pCRISPRyl with MFE1 targeting sgRNA	Schwartz et al., 2017b
pCRISPRyl_PAH1	pCRISPRyl with PAH1 targeting sgRNA	This study
pCRISPRyl_GSY1	pCRISPRyl with GSY1 targeting sgRNA	This study
**Strains**
PO1f	MatA, leu2-270, ura3-302, xpr2-322, axp1-2	Madzak et al., 2000
HEBI-L-U	PO1f UAS1B8-TEF-HMG1-CYC::D17 UAS1B8-TEF-CrtE-CYC::A08 UAS1B8-TEF-CrtB-CYC::AXP UAS1B8-TEF-CrtI-CYC::XPR2	Schwartz et al., 2017b
HEBI ΔMFE1	PO1f HEBI ΔMFE1	This study
HEBI ΔGSY1	PO1f HEBI ΔGSY1	This study
HEBI ΔPAH1	PO1f HEBI ΔPAH1	This study
HEBI-L	PO1f HEBI +*URA3*	This study
HEBI-U	PO1f HEBI +*LEU2*	This study
HEBI	PO1f HEBI +*URA3* +*LEU2*	This study
HEBI ERG10	PO1f HEBI +*ERG10*	This study
HEBI ERG13	PO1f HEBI +*ERG13*	This study
HEBI ERG20	PO1f HEBI +*ERG20*	This study
HEBI HMG1	PO1f HEBI +*HMG1*	This study
HEBI MVD1	PO1f HEBI +*MVD1*	This study
HEBI IDI1	PO1f HEBI +*IDI1*	This study
HEBI CrtE	PO1f HEBI +*CrtE*	This study
HEBI CrtB	PO1f HEBI +*CrtB*	This study
HEBI CrtI	PO1f HEBI +*CrtI*	This study
HEBI HV	PO1f HEBI *HMG1*::LEU2 *MVD1*::E07	This study
HEBI HV8	PO1f HEBI *HMG1*::LEU2 *MVD1*::E07 +*ERG8*	This study
HEBI HHV	PO1f HEBI *HMG1*::LEU2 *MVD1*::E07 +*HMG1*	This study
HEBI HVI	PO1f HEBI *HMG1*::LEU2 *MVD1*::E07 +*CrtI*	This study
HEBI HV8I	PO1f HEBI *HMG1*::LEU2 *MVD1*::E07 +*ERG8* +*CrtI*	This study

### Plasmid Design and Construction

Native genes in the mevalonate pathway were obtained via PCR amplification from purified genomic DNA of the PO1f strain. Genomic DNA was isolated using the Zymo Research YeaStar genomic DNA kit. Genes were amplified using Phusion DNA Polymerase with primers listed in Supplementary Table S1. Amplified genes were cloned into pUAS1B8-TEF(136)-hrGFP (Blazeck et al., 2011) digested with BssHII and NheI by Gibson Assembly yielding plasmids as shown in Supplementary Figure S1. As such, all overexpressed genes had the same promoter, the UAS1B8-TEF(136) (Supplementary Table S2). Plasmid construction for markerless integration was done as previously described, with all genes again using the UAS1B8-TEF(136) promoter (Schwartz et al., 2017b). All cloning enzymes were purchased from New England Biolabs.

### Strain Engineering and Genome Editing

Overexpression of genes in the mevalonate and lycopene pathways were carried out with linear DNA transformations using the selectable leucine marker (LEU2). Plasmids containing LEU2 and a gene of interest, derived from pIW209, were linearized with the restriction enzyme KpnI (Supplementary Figure S1). The linearized DNA was column purified using Zymo Research Clean & Concentrator. The DNA was then transformed into the yeast strain HEBI-L (**Table 1**) and screened by plating the transformants on minimal media agar plates (SD -Leu -Ura). Uracil auxotrophy was alleviated using an analogous methodology, with plasmids derived from pIW245. Transformation of linear DNA resulted in integration of the fragment into a random location in the genome. Transformations were conducted at stationary phase and followed a previously described procedure (Schwartz et al., 2017b). After 2 days of outgrowth at 30°C, healthy colonies were selected and used for lycopene characterization. Markerless integration enabled by CRISPR-Cas9 and homologous repair donors was used for genes identified as limiting in the mevalonate and lycopene pathways to facilitate the overexpression of more than 2 genes (Schwartz et al., 2017b). Gene disruptions were done in the HEBI-L-U strain using CRISPR-Cas9 with repair by nonhomologous end-joining, and auxotrophies alleviated after.

### Culture Conditions

Cultivation of carotenoid producing yeast strains was conducted in 250 mL baffled Erlenmeyer shake flask. Strains were initially grown in YPD medium and cultured overnight at 30°C. Flasks containing 25 mL of YPD10 were then inoculated to an OD600 of 0.1 and grown in a 30°C shaker at 200 RPM. Aliquots of the shake flask cultures were taken on the 4th day and used for DCW and lycopene quantification. Extended cultures (12-day cultures) were carried out in a similar manner, with DCW not taken until the last day of the trial and glucose added via pipetting at 2 days intervals by addition of 2.5 mL of 40% glucose.

Bioreactor experiments were performed in a 1-L batch reactor (Biostat A, Sartorius, Supplementary Figure S2). The bioreactor enabled control of dissolved oxygen (25%, maintained by constant aeration with oxygen and air, and variable stir rates), temperature (30°C, maintained through a heat jacket), and pH (6.8, maintained through automated addition of 3 M NaOH). Starter cultures were grown overnight in YPD and used to inoculate 500 mL of media to an OD600 of 0.02. At 24 h intervals, 25 mL of 40% glucose was added. At each 24 h interval, 10 mL was removed and used to quantify the specific lycopene content of the culture.

### Lycopene Quantification

Extraction of carotenoids followed the method provided by Chen and coworkers with a few modifications (Chen et al., 2016; Schwartz et al., 2017b). Briefly, a 5 mL sample was taken and used for dry cell weight (DCW), and a 1 mL sample was used for extraction of lycopene by centrifuging at 5,000 *g* for 3 min, washing the cell pellet with water, resuspending in 1 mL 3M HCl, and incubating at 100°C for 2 min. Cells were then cooled in an ice bath for 3 min, washed with water, and resuspended in 1 mL of acetone. Two hundred-μL of 500–750 μm glass beads (Fisher Scientific) were then added, and the mixture was vortexed for 2 min. Subsequently, the mixture was centrifuged and the supernatant was subjected to analysis for lycopene. Lycopene was quantified by measuring absorbance at 472 nm and compared to a standard curve of purchased lycopene (Sigma–Aldrich, Supplementary Figure S3).

### Statistical Analysis

**FIGURE 2:**
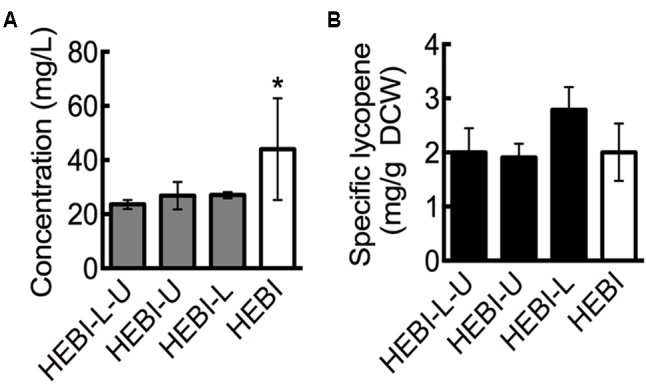
Effect of leucine and uracil auxotrophies on lycopene production. **(A)** Lycopene titer for strains with and without leucine and uracil auxotrophies. **(B)** Specific lycopene content (mg lycopene/g DCW) for strains with and without leucine and uracil auxotrophies. Strains were grown for 4 days in 25 mL of YPD10 at 30°C in baffled shake flasks. Dry cell weight produced by each strain: HEBI-L-U 2.4 ± 0.4 g/L, HEBI-U 2.8 ± 0.2 g/L, HEBI-L 2.0 ± 0.4 g/L, and HEBI 4.3 ± 0.8 g/L. Statistical significance from the HEBI-L-U strain is indicated by “^∗^”. HEBI indicates the following overexpressions in the PO1f background: H = HMG1, E = CrtE, B = CrtB, I = CrtI.

**FIGURE 3:**
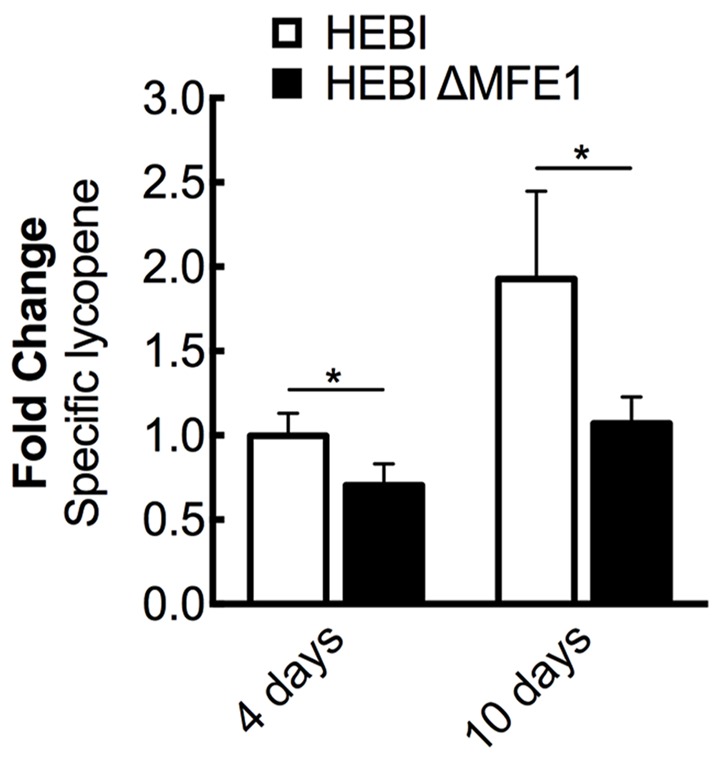
The effect of β-oxidation on lycopene production in *Y. lipolytica* PO1f. Relative specific lycopene content of strains with and without intact β-oxidation at 4 and 10 days of culture time. β-oxidation was disrupted by knockout of *MFE1*. Strains were grown for 4 and 10 days in 25 mL of YPD10 at 30°C in baffled shake flasks. Bars and error bars represent the mean and standard deviation, respectively, of biological triplicates. Statistical significance from the HEBI strain is indicated by “^∗^”. At 4 days, the HEBI strain produced 2.8 ± 0.3 mg lycopene/g DCW.

**FIGURE 4:**
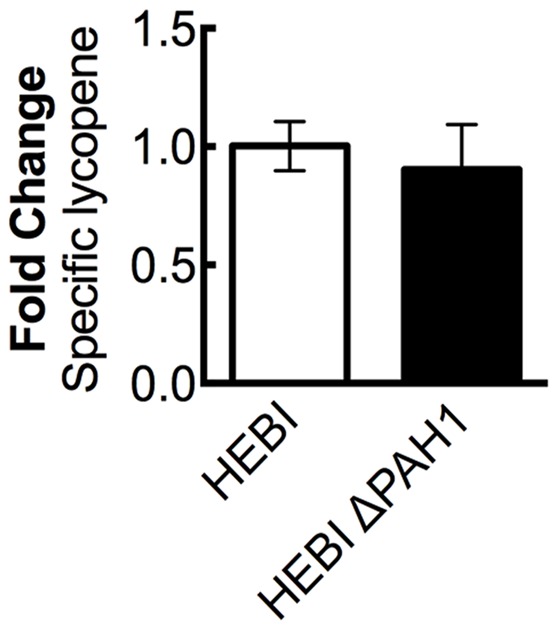
The effect of *PAH1* knockout on lycopene production in *Y. lipolytica* PO1f. Relative specific lycopene content of strains with and without disruption of *PAH1*. Strains were grown for 4 days in 25 mL of YPD10 at 30°C in baffled shake flasks. Bars and error bars represent the mean and standard deviation, respectively, of biological triplicates. Statistical significance from the HEBI strain is indicated by “^∗^”. No statistically significant difference was detected. The HEBI strain produced 5.3 ± 0.3 mg lycopene/g DCW.

**FIGURE 5:**
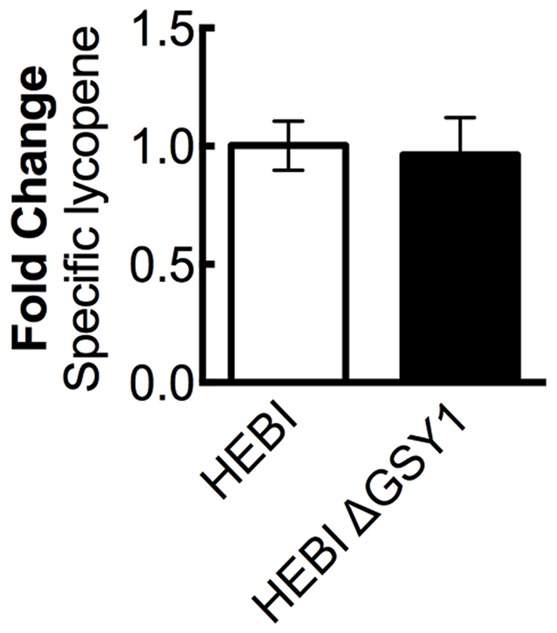
The effect of disrupting glycogen biosynthesis on lycopene production in *Y. lipolytica* PO1f. Relative specific lycopene content of strains with and without intact glycogen biosynthesis. Strains were grown for 4 days in 25 mL of YPD10 at 30°C in baffled shake flasks. Bars and error bars represent the mean and standard deviation, respectively, of biological triplicates. Statistical significance from the HEBI strain is indicated by “^∗^”. The HEBI strain produced 3.4 ± 0.2 mg lycopene/g DCW.

**FIGURE 6:**
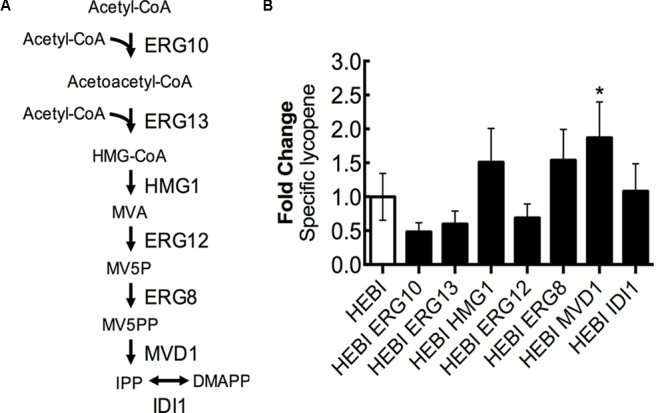
Engineering mevalonate biosynthesis pathway. **(A)** Schematic representation of the mevalonate pathway. **(B)** Relative specific lycopene production from strains with a single gene in the mevalonate pathway overexpressed. Strains were grown for 4 days in 25 mL of YPD10 at 30°C in baffled shake flasks. Bars and error bars represent the mean and standard deviation, respectively, of biological triplicates. Statistical significance from the HEBI strain is indicated by “^∗^”. The HEBI strain produced 3.2 ± 0.9 mg lycopene/g DCW.

**FIGURE 7:**
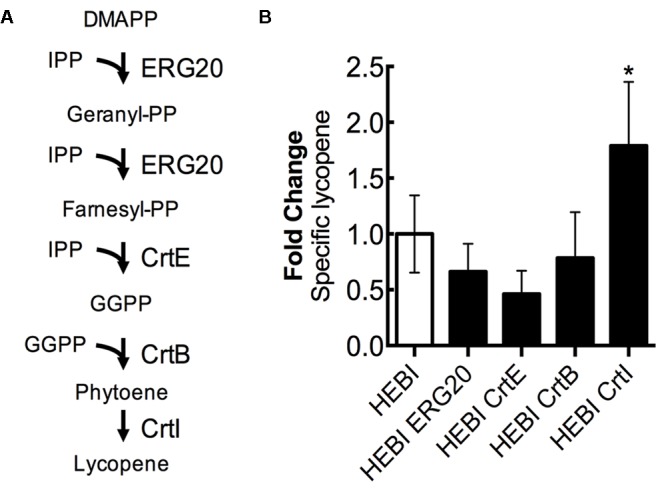
Engineering the lycopene biosynthetic pathway. **(A)** Schematic representation of the lycopene biosynthetic pathway. **(B)** Relative specific lycopene production from strains with a single gene in the lycopene biosynthetic pathway overexpressed. Strains were grown for 4 days in 25 mL of YPD10 at 30°C in baffled shake flasks. Bars and error bars represent the mean and standard deviation, respectively, of biological triplicates. Statistical significance from the HEBI strain is indicated by “^∗^”. The HEBI strain produced 3.2 ± 0.9 mg lycopene/g DCW.

**FIGURE 8:**
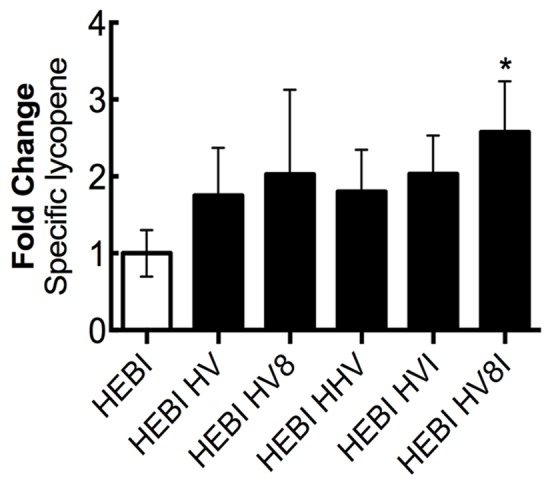
Combing successful overexpressions to optimize lycopene production. Relative specific lycopene of strains combining successful engineering strategies to maximize lycopene production in 4-day shake flask cultures. Strains were grown for 4 days in 25 mL of YPD10 at 30°C in baffled shake flasks. Bars and error bars represent the mean and standard deviation, respectively, of biological triplicates. Statistical significance from the HEBI strain is indicated by “^∗^”. The HEBI strain produced 2.0 ± 0.5 mg lycopene/g DCW. HV8I indicates the following overexpressions in the HEBI background: H = HMG1, V = MVD1, 8 = ERG8, I = CrtI. Names with multiple copies of a given letter indicate multiple overexpressions of the indicated gene.

Experiments were performed in triplicate, and the mean and standard deviation are reported. Comparisons between two means were done using a two-tailed *T*-test (**Figures 3–5**), while comparisons among three or more means was done using 1-way ANOVA with *post hoc* Fisher’s analysis (**Figures 2, 6B, 7B, 8**). A p-value less than 0.05 was used to determine statistical significance.

## Results

### Effect of Auxotrophy on Growth and Lycopene Production

The initial lycopene producing strain used here, HEBI-L-U (see **Table 1**), was generated in a previous study by using a CRISPR-Cas9-enabled markerless gene integration strategy (Schwartz et al., 2017b). As such, the strain retained both the *ura3* and *leu2* auxotrophies that were present in the starting *Y. lipolytica* PO1f strain. To investigate the effect of alleviating these auxotropies, we sought to determine the effect of restoring functional *LEU2* and *URA3* genes on lycopene production.

Linear DNA containing one of the selectable markers was transformed into the HEBI-L-U and randomly integrated into the genome to alleviate each of the auxotrophies. Lycopene production from the resulting strains, reported as titer, is presented in **Figure 2A**. As shown, alleviating either auxotrophy individually did not have an effect on lycopene titer, while alleviating both increased lycopene titer from 23 ± 1.6 mg/L to 44 ± 18.8 mg/L, an almost two-fold increase. This increase was primarily due to higher cell mass production. When lycopene production was normalized to DCW to give the specific lycopene yield (mg lycopene/g DCW), the HEBI-L-U and HEBI strains were almost identical, with both producing approximately 2 mg lycopene/g DCW (**Figure 2B**). Alleviating uracil and leucine auxotrophies resulted in higher overall cell mass and a corresponding increase in lycopene production, but did not affect the specific lycopene content.

### Disruption of β-Oxidation

A previously demonstrated strategy to increase carotenoid production in *Y. lipolytica* is the disruption of β-oxidation (Matthaus et al., 2014). β-oxidation is the biological process through which intracellular lipids are degraded and metabolized. As sequestration of lycopene in lipid droplets (which are composed primarily of neutral lipids) has been reported to be important for overall production of lycopene, blocking this degradation pathway may increase lycopene accumulation. To test this hypothesis in our host, we used a previously described CRISPR-Cas9 system to disrupt the *MFE1* gene in the HEBI-L-U strain, yielding HEBI ΔMFE1. Transformation with pCRISPRyl_MFE1, outgrowth for 2 days, and plating on rich media enabled the identification of a strain containing an indel mutation in the *MFE1* gene that rendered it non-functional (Supplementary Table S3).

The HEBI and HEBI ΔMFE1 strains were grown for 4 days and their respective lycopene yields were measured (**Figure 3**). The HEBI strain was found to have higher lycopene production than the HEBI ΔMFE1 strain, with HEBI ΔMFE1 yielding a 30% reduction in specific lycopene content. The reduction in lycopene yield was more apparent when the cultures were grown to 10 days, when the HEBI strain produced 1.8-fold more lycopene than HEBI ΔMFE1. Disruption of β-oxidation appeared to have a deleterious effect on specific lycopene content, especially at extended culture times once glucose is exhausted.

### Increasing Membrane Availability for Enhanced Pathway Biocatalysis

A previous study in *Y. lipolytica* found that disruption of phosphatidic acid phosphohydrolase, *PAH1*, resulted in increased expression of proteins localized to the ER (Guerfal et al., 2013). Several genes needed for synthesis of lycopene, including HMG1, are localized to the ER, and so increasing membrane protein expression might increase flux to lycopene. To test this hypothesis, the *PAH1* gene was disrupted in the HEBI-L-U strain by CRISPR-Cas9, with an indel mutation rendering the *PAH1* gene non-functional (Supplementary Table S3). Lycopene production between the HEBI and HEBI ΔPAH1 strains were compared; however, no significant change in lycopene production was observed (**Figure 4**).

### Disrupting Glycogen Biosynthesis to Divert Flux to Lycopene

A recent work showed that disruption of glycogen synthase (*GSY1*) in *Y. lipolytica* gave an increase in lipid accumulation by diverting carbon from carbohydrate storage to lipid storage (Bhutada et al., 2017). We hypothesized that eliminating glycogen biosynthesis may increase overall acetyl-CoA production, and that some of this increased pool of acetyl-CoA might then enter the mevalonate pathway and lead to higher lycopene yields. The *GSY1* gene was disrupted in the HEBI-L-U strain by CRISPR-Cas9 (Supplementary Table S3) and HEBI and HEBI ΔGSY1 were compared for lycopene production (**Figure 5**). It was found that under the conditions used in this study, disruption of *GSY1* did not lead to an increase in lycopene production.

### Identifying Limiting Steps in Mevalonate Biosynthesis

Lycopene biosynthesis is dependent upon the products of the mevalonate pathway as precursors, and so increasing flux through the mevalonate pathway is likely to increase lycopene production. The mevalonate pathway, from acetyl-CoA to isopentenyl diphosphate (IPP), consists of 6 enzymatic reactions (**Figure 6A**). First, 2 molecules of acetyl-CoA are condensed to form acetoacetyl-CoA by acetoacetyl-CoA thiolase (ERG10). A third acetyl-CoA molecule is then added to form 3-hydroxy-3-methylglutaryl-CoA (HMG-CoA) by HMG-CoA synthase (ERG13). The next step, which is reported to be rate-limiting (Donald et al., 1997), involves the conversion of HMG-CoA to mevalonate (MVA) by HMG-CoA reductase (HMG1) while also consuming 2 molecules of NADPH. Mevalonate is then phosphorylated twice, first by mevalonate kinase (ERG12) and second by phosphomevalonate kinase (ERG8), with each phosphorylation consuming 1 molecule of ATP and resulting in mevalonate-5-diphosphate (MV5PP). MV5PP is then converted to IPP by mevalonate pyrophosphate decarboxylase (MVD1), which consumes 1 molecule of ATP and removes 1 carbon from the molecule. Finally, IPP can be isomerized to dimethylallyl-pyrophosphate (DMAPP) by isopentenyl diphosphate:dimethylallyl diphosphate isomerase (IDI1).

To identify the limiting step(s) in this pathway in the context of lycopene biosynthesis, the HEBI-L-U strain was separately transformed with an expression cassette for each gene in the pathway and the auxotrophies were alleviated (**Figure 6B**). Production of lycopene with each gene separately overexpressed was then measured. Only one gene showed a statistically significant increase in lycopene production, the gene encoding MVD1. MVD1 overexpression yielded an increase of ∼1.9-fold in specific lycopene yield. Two additional genes, ERG8 and HMG1, appeared to increase lycopene production, although the results were not statistically significant (p∼0.1). ERG10, ERG13, and ERG12 appeared to reduce specific lycopene content (but, the reductions are not statistically significant), possibly due to the overexpressions leading to the accumulation of intermediates and an unbalanced pathway.

### Identifying Limiting Steps in the Lycopene Biosynthesis Pathway

For the purposes of this discussion, we describe the lycopene biosynthetic pathway as the steps necessary to convert the products of the mevalonate pathway (IPP and DMAPP) to lycopene (**Figure 7A**). This pathway is a combination of native enzymes and heterologous activities, as *Y. lipolytica* does not natively synthesize lycopene. First, DMAPP and IPP are condensed to form geranyl-diphosphate (geranyl-PP) by farnesyl pyrophosphate synthetase (ERG20). The same enzyme, ERG20, then catalyzes the addition of a second IPP molecule to produce farnesyl-diphosphate (farnesyl-PP). Farnesyl-PP can then be converted to geranylgeranyl-diphosphate (GGPP) by geranylgeranyl diphosphate synthase (BTS1). In a previous work, we found that a heterologous geranylgeranyl diphosphate synthase (CrtE, from *Pantoea ananatis*) was more effective (Schwartz et al., 2017b), and so it is used exclusively in this work. The production of GGPP is the end of the native yeast biosynthetic pathway, the subsequent steps are heterologous. Two molecules of GGPP are condensed by phytoene synthase (CrtB, from *P. ananatis*) to form phytoene. Phytoene then undergoes 4 consecutive desaturation reactions catalyzed by lycopene synthase (CrtI, from *P. ananatis*) to yield lycopene.

Each enzyme in this pathway was separately overexpressed by random integration in the HEBI-L-U strain, and the auxotrophies were alleviated. The lycopene production from each strain was quantified (**Figure 7B**). Only 1 gene showed an increase in lycopene production relative to the HEBI strain: specific lycopene production was increased 1.8-fold in the strain with a second copy of CrtI. These results suggested that the final step, four consecutive desaturations of phytoene, is the limiting step in lycopene biosynthesis. ERG20, CrtE, and CrtB appeared to reduce specific lycopene content, possibly due to intermediate accumulation; however, none of the observed reductions in lycopene were statistically significance.

### Combination of Successful Strategies to Maximize Lycopene Production

Through sequentially testing a variety of different genetic knockouts and pathway enzyme overexpressions, a set of changes to maximize lycopene production was identified. Alleviation of both the *ura3* and *leu2* auxotrophies was combined with sequential overexpressions of different combinations of the mevalonate and lycopene pathway enzymes that resulted in higher production (HMG1, ERG8, MVD1, and CrtI; **Figure 8**). To achieve this, HMG1 and MVD1 were integrated into the genome using CRISPR-Cas9-mediated markerless integration to yield HEBI HV -L-U. The gene coding for HMG1 was integrated into the disrupted *LEU2* locus in the PO1f genome, and the gene for MVD1 was integrated in a similar manner into the pseudogene YALI0E07645g locus. Integration was confirmed by PCR. The auxotrophies of this strain were then alleviated to yield HEBI HV. Linear expression cassettes for ERG8 and CrtI overexpression were transformed into the HEBI HV -L-U strain to yield HEBI HV8 and HEBI HVI, respectively. Due to literature reports of HMG1 being limiting, a third exogenous copy of HMG1 was also integrated in the same way, yielding HEBI HHV. Finally, linear DNA fragments containing the URA3 and LEU2 selectable markers and ERG8 and CrtI were separately transformed into HEBI HV -L-U, to yield HEBI HV8I. Using this strategy, the quadruple integration gave the highest level of lycopene production, at about 2.6-fold higher than the HEBI strain. All of the triple and double integrations produced similar specific lycopene contents, indicating that the quadruple integrant had a synergistic effect to maximize flux to lycopene.

**FIGURE 9:**
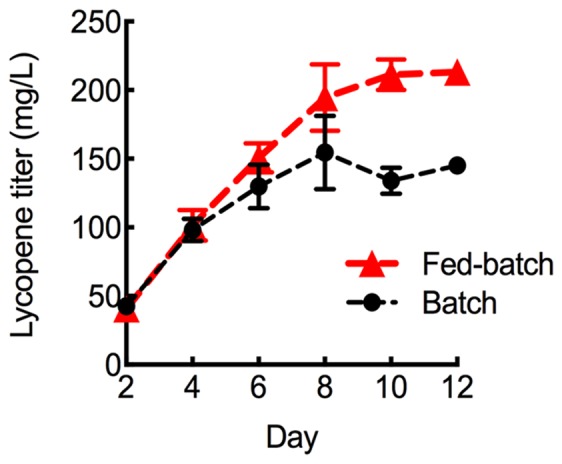
Lycopene production in fed-batch shake flask cultures. Strains were grown for 12 days in 25 mL of YPD10 at 30°C in baffled shake flasks with glucose feeding as described. Bars and error bars represent the mean and standard deviation, respectively, of biological triplicates.

To further maximize lycopene production, the HEBI HV8I strain was grown to longer times. **Figure 3** demonstrates a 2-fold increase in specific lycopene content when increasing the culture time from 4 to 10 days. We tested to see if this would be replicated in the HEBI HV8I strain. Additionally, a glucose feeding strategy was tested. For glucose feeding, every 2 days 2.5 mL of 40% glucose was added to the culture of HEBI HV8I in YPD10 media. For both feeding and non-feeding cultures, samples were taken every 2 days to determine the titer of lycopene. The results are shown in **Figure 9**. As shown, no feeding of glucose resulted in a lycopene titer of 155 ± 26 mg/L, while the glucose feeding strategy maximized lycopene titer throughout the culture and at day 12, reaching as high as 213 ± 8 mg/L lycopene.

**FIGURE 10:**
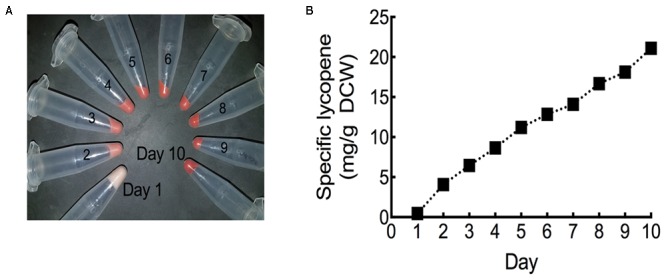
Lycopene production in fed-batch bioreactor. **(A)** Cell pellets taken from each day of bioreactor culture post-inoculation. Red coloration is due to lycopene production. **(B)** Specific lycopene production at each day of bioreactor culture post-inoculation. Data represents technical triplicates of a single bioreactor experiment.

In order to fully maximize specific lycopene yields, the HEBI HV8I strain was cultured in a fed batch bioreactor. Specific lycopene content measurements and cell pellet images were taken each day over the 10-day experiment. As can be seen in **Figure 10A** and Supplementary Figure S2, the culture turned visibly red, indicating significant lycopene production. **Figure 10B** shows specific lycopene measured over the course of the culture. After 10 days of growth, a specific lycopene yield of 21.1 mg/g DCW was obtained.

## Discussion

The HEBI-L-U strain of *Y. lipolytica* that had previously been generated (Schwartz et al., 2017b) produced a specific lycopene content of 2 mg/g DCW when grown in shake flasks for 4 days in YPD10 media. Alleviation of both the *leu2* and *ura3* auxotrophies did not result in increased specific lycopene content, but did result in a 1.9-fold increase in lycopene titer, from 23 mg/L to 44 mg/L. It has been shown elsewhere that the presence of auxotrophies can cause an organism to grow more slowly than the equivalent prototroph on rich media (Pronk, 2002), the results presented here are in agreement with this report. The increased production by eliminating the *leu2* auxotrophy also agrees with a previous work in lipid production in *Y. lipolytica*, which found that leucine-mediated signaling was important for efficient lipogenesis (Blazeck et al., 2014). This role of leucine-mediated signaling may also have caused the reduced DCW production by strain HEBI-L relative to HEBI-L-U and HEBI-U.

It has been hypothesized that lipid droplets are able to sequester intracellularly synthesized carotenoids and thus allow *Y. lipolytica* to avoid insertion of carotenoids into cellular and organellar membranes, which can be harmful to the cell (Sung et al., 2007). Based on the results shown in **Figure 3**, disruption of β-oxidation (an oxidative degradation pathway for storage lipids found in lipid droplets) appears to be counter-productive for increasing lycopene production under the conditions used at extended culture times. This is in contrast to a previous study, which found as much as a three-fold increase in lycopene production following disruption of β-oxidation (Matthaus et al., 2014). The discrepancy is likely due to a difference in the culture conditions. It is also possible that different gene knockouts to disrupt β-oxidation might have different effects. For example, Matthaus and coworkers disrupted POX1-6, while this work disrupted MFE1. Our finding of increasing levels of lycopene at longer time points may be due to degradation of lipids (via β-oxidation) leading to production of acetyl-CoA, which then enters the mevalonate pathway and is converted to lycopene. It is also possible that the conditions used in this study (10% glucose) allowed for more than enough lipids to be produced, and so some degradation of lipid droplets is not harmful. The balance between carotenoid production and lipid droplet size and capacity for sequestration is an open question that merits further study.

Two other strategies were attempted for engineering *Y. lipolytica* as a host for lycopene production. The first was an attempt to increase ER-bound enzyme expression, as two key native enzymes are ER-bound, HMG1 and ERG20. To increase expression of these enzymes, we sought to increase the total area of the ER by *PAH1* disruption (Guerfal et al., 2013). However, no increase in lycopene production was detected. This suggests that the natively available ER is adequate for HMG1 expression, or that disruption of *PAH1* has a negative effect on the host cell’s metabolism. It has also been reported to decrease lipid droplet size, which could prevent lycopene from being sequestered effectively.

The second strategy, builds from a recent study that examined the effects glycogen production on lipid synthesis by disrupting *GSY1*. The study found that in *Y. lipolytica*, up to 16% of the cell mass could be glycogen, and that eliminating glycogen production forced carbon flux to lipids, resulting in a 60% increase in lipids (Bhutada et al., 2017). As increased lipid production indicates higher flux through the metabolic acetyl-CoA node, we hypothesized some of this acetyl-CoA would enter the mevalonate pathway and be converted to lycopene. However, no significant increase in lycopene production was detected. This could be due to a limitation of enzyme activity for flux down the mevalonate pathway, or could be due to the use of different culture conditions.

In investigating the effect of overexpression of each step in the mevalonate and lycopene pathways, four genes were identified that appeared to increased lycopene production. HMG1 is commonly known to be the bottleneck step for flux down the mevalonate pathway in yeast, and has thus been targeted for overexpression in a number of previous studies in both *S. cerevisiae* and *Y. lipolytica*, with consistent success (Yan et al., 2012; Matthaus et al., 2014; Gao S.L. et al., 2017). Commonly, a truncated version of HMG1 is used, with the membrane localization and regulatory regions removed. In this study, however, the full length HMG1 was used for overexpression. The final step in carotenoid production (CrtI in this study) was identified as limiting in *Y. lipolytica* when producing β-carotene, and increasing the number of overexpression cassettes gave increased levels of production (Gao S.L. et al., 2017). In this work, integrating a second copy of the CrtI gene for additional overexpression produced a 1.8-fold increase in lycopene biosynthesis (**Figure 7B**), indicating that the final conversion step is partially limiting. A previous study tested overexpression of ERG8 and MVD1 for production of β-carotene in *Y. lipolytica*, but found that neither resulted in an increase in β-carotene production when separately overexpressed (Gao S.L. et al., 2017). Here, overexpression of a second copy of ERG8 increased lycopene production by 1.5-fold and overexpression of MVD1 increased lycopene production by 1.9-fold (**Figure 6B**). The difference in these results is likely due to the difference in the background strains. In the β-carotene study, the strain already produced around 20 mg β-carotene/g DCW, while in our study the base strain was not as strong of a carotenoid producer, making only 3.2 mg lycopene/g DCW.

To further increase lycopene production, cells containing all identified beneficial genetic modifications were grown continuously for 12 days in shake flasks and again in a 10-day, fed-batch bioreactor experiment. Growth in shake flasks produced upward of 9.8 mg lycopene/g DCW, while the 10-day bioreactor experiment resulted in a specific lycopene content of 21.1 mg lycopene/g DCW. To our knowledge, the specific lycopene yield achieved in our bioreactor culture is the highest reported value for *Y. lipolytica*, a 1.3-fold improvement over the previously report 16 mg lycopene/g DCW achieved in a similar bioreactor experiment (Matthaus et al., 2014). However, higher yields have been achieved in other hosts including 55.6 mg/g DCW in *S. cerevisiae* (Chen et al., 2016). The study in *S. cerevisiae* was able to achieve such high titers by combining 4 knockouts of genes with roles not directly involved in the mevalonate and lycopene biosynthetic pathways (using the *Saccharomyces* knockout collection), and by testing different mating types. Further increases in titer in *Y. lipolytica* may be achievable by using strategies demonstrated in other yeast metabolic engineering studies. For example, using a truncated form of HMG1 and overexpressing additional copies would likely increase flux through the mevalonate pathway (Gao S.L. et al., 2017). Reduction of FPP flux to the squalene biosynthetic pathway (a competing pathway for carotenoid production) has also shown to increase production in yeast (Xie et al., 2015); however, one study showed that this strategy was not successful in *Y. lipolytica* (Gao S.L. et al., 2017). Adaptive laboratory evolution with oxidative stress selection has resulted in increased carotenoid production in *S. cerevisiae* (Reyes et al., 2014) – an analogous strategy may be able to increase lycopene production in *Y. lipolytica*.

## Conclusion

Several different host cell engineering strategies were tested for enhanced lycopene biosynthesis in *Y. lipolytica*. Alleviating leucine and uracil auxotrophies was found to increase cell growth, and therefore lycopene titers. Disruption of β-oxidation to prevent lipid degradation, disruption of *PAH1* to increase ER-membrane proliferation, and disruption of glycogen synthesis were all tested for their effect on lycopene yields; however, none of these strategies significantly increase lycopene production in the engineered strains of *Y. lipolytica* PO1f used here. Individual overexpressions of each enzymatic step from acetyl-CoA to lycopene were analyzed, with 4 enzymes successfully increasing lycopene production: HMG1, MVD1, ERG8, and CrtI. By combining the beneficial engineering strategies, extending culture time, and culturing under fed-batch conditions lycopene titer reached 213 mg/L. This systematic strain engineering presented here, provides both biological insights into carotenoid production and a roadmap for future terpenoid engineering studies in *Y. lipolytica*.

## Author Contributions

CS and IW planned the experiments and conceived the idea. KF, CS, and JM performed the experiments. KF, CS, and IW analyzed the data and wrote the manuscript with editing and input from all authors.

## Conflict of Interest Statement

The authors declare that the research was conducted in the absence of any commercial or financial relationships that could be construed as a potential conflict of interest.
