# Supplementary material for: Host and Pathway Engineering for Enhanced Lycopene Biosynthesis in Yarrowia lipolytica
Source: Front Microbiol. 2017 Nov 20;8:2233. doi: 10.3389/fmicb.2017.02233 (PMC5727423; doi:10.3389/fmicb.2017.02233)
Supplement: Supplementary file 1 [file Data_Sheet_1.DOCX]

*Supplementary Material*

Host and Pathway Engineering for Enhanced Lycopene Biosynthesis in *Yarrowia lipolytica*

Cory Schwartz^†1^, Keith Frogue^†1^, Joshua Misa^1^, Ian Wheeldon^1^*

^†^Contributed equally

Affiliations

^1^Chemical and Environmental Engineering, University of California, Riverside, California 92521, United States

**Table S1:** Primers used in this study

**Table S2:** Sequences of non-native parts used

**Table S3:** Sequencing of disrupted genes

**Figure S1:** Map of linear integration plasmid for HMG1

**Figure S2:** Bioreactor images

**Figure S3.** Absorbance spectrum of lycopene

**Table S1.** Primers used in this study

| **Primer** | **Sequence** | **Use** |
| --- | --- | --- |
| Cr_793 | TCTGAGTATAAGAATCATTCAAAGGCGCGCATGCGACTCACTCTGCCCC | ERG10 cloning |
| Cr_794 | GCGTGACATAACTAATTACATGAGGCTAGCCTACTCGACAGAAGAGACCTTCTTGATG | ERG10 cloning |
| Cr_795 | TCTGAGTATAAGAATCATTCAAAGGCGCGCATGTCGCAACCCCAGAACG | ERG13 cloning |
| Cr_796 | GCGTGACATAACTAATTACATGAGGCTAGCCTACTGCTTGATCTCGTACTTTCGTCG | ERG13 cloning |
| Cr_797 | TCTGAGTATAAGAATCATTCAAAGGCGCGCATGTCCAAGGCGAAATTCGAAAG | ERG20 cloning |
| Cr_798 | GCGTGACATAACTAATTACATGAGGCTAGCCTACTTCTGTCGCTTGTAAATCTTGG | ERG20 cloning |
| Cr_985 | TCTGAGTATAAGAATCATTCAAAGGCGCGCATGCTACAAGCAGCTATTGGAAAG | HMG1 cloning |
| Cr_986 | GCGTGACATAACTAATTACATGAGGCTAGCCTATGACCGTATGCAAATATTCGAAC | HMG1 cloning |
| Cr_987 | TCTGAGTATAAGAATCATTCAAAGGCGCGCTGGCCATCTTCGCCGAG | CrtE cloning |
| Cr_988 | GCGTGACATAACTAATTACATGAGGCTAGCTTATCCCACCATGGCCAGCTTC | CrtE cloning |
| Cr_989 | TCTGAGTATAAGAATCATTCAAAGGCGCGATGAACAACCCCTCTCTGC | CrtB cloning |
| Cr_990 | GCGTGACATAACTAATTACATGAGGCTAGCTACAGGGGTCGCTGC | CrtB cloning |
| Cr_991 | TCTGAGTATAAGAATCATTCAAAGGCGCGATGAAGCCCACCACCG | CrtI cloning |
| Cr_992 | GCGTGACATAACTAATTACATGAGGCTAGCTAGATCAGGTCCTCCAGCATC | CrtI cloning |
| Cr_999 | TCTGAGTATAAGAATCATTCAAAGGCGCGCATGGACTACATCATTTCGGCGC | ERG12 cloning |
| Cr_1000 | GCGTGACATAACTAATTACATGAGGCTAGCCTAATGGGTCCAGGGACCGATGT | ERG12 cloning |
| Cr_1001 | TCTGAGTATAAGAATCATTCAAAGGCGCGCATGATCTTCGACCGGCTCTTC | ERG8 cloning |
| Cr_1002 | GCGTGACATAACTAATTACATGAGGCTAGCCTACTTGAACCCCTTCTCGAGC | ERG8 cloning |
| Cr_1003 | TCTGAGTATAAGAATCATTCAAAGGCGCGCATGATCCACCAGGCCTCCAC | MVD1 cloning |
| Cr_1004 | GCGTGACATAACTAATTACATGAGGCTAGCCTACTTGCTGTTCTTCAGAGAACCATCCTC | MVD1 cloning |
| Cr_1005 | TCTGAGTATAAGAATCATTCAAAGGCGCGCATGACGACGTCTTACAGCGACAAAAT | IDI1 cloning |
| Cr_1006 | GCGTGACATAACTAATTACATGAGGCTAGCCTACTTGATCCACCGCCGAATCT | IDI1 cloning |
| Cr_370 | GCTCAAGTTCTCTCTTAACATGAAGC | Leu2 integration |
| Cr_371 | CAATAGAACCTGTTTTGAGGCTGAAAC | Leu2 integration |
| Cr_419 | CTCGAAGGCTTTAATTTGCcctagg | CYC integration |
| Cr_549 | GACCCGCACCTCTAATTATAAGACC | XDH integration |
| Cr_550 | GGCTTCCGCTCTGTGGTAC | XDH integration |
| Cr_100 | GGGTCGGCGCAGGTTgacgtGCTCTGAACGACTCTAAACAGTTTTAGAGCTAGAAATAGC | PAH1 CRISPR |
| Cr_101 | GCTATTTCTAGCTCTAAAACTGTTTAGAGTCGTTCAGAGCacgtcAACCTGCGCCGACCC | PAH1 CRISPR |
| Cr_137 | ACCGAGCTGCCCATGAAGG | PAH1 screening |
| Cr_138 | GGCTCTGTAGGCTCTGTGAGC | PAH1 screening |
| Cr_102 | GGGTCGGCGCAGGTTGACGTGCTGTTCGAGGTCGCCACCGGTTTTAGAGCTAGAAATAGC | GSY1 CRISPR |
| Cr_103 | GCTATTTCTAGCTCTAAAACCGGTGGCGACCTCGAACAGCACGTCAACCTGCGCCGACCC | GSY1 CRISPR |
| Cr_1176 | CAAAGTGACTGTACTTGAACACTG | GSY1 screening |
| Cr_1177 | GACTTTAGTGTTTTGTTGATGGCTTC | GSY1 screening |
| Cr_084 | CAAGGCTGTTGCTAACTACGACTC | MFE1 screening |
| Cr_804 | TGTCGTCGCCCTTGAAAACAG | MFE1 screening |

**Table S2.** Sequences of non-native parts

| Part | Sequence |
| --- | --- |
| UAS1B8-TEF(136) | GAATTCCTGAGGTGTCTCACAAGTGCCGTGCAGTCCCGCCCCCACTTGCTTCTCTTTGTGTGTAGTGTACGTACATTATCGAGACCGTTGTTCCCGCCCACCTCGATCCGGTCTAGACTGAGGTGTCTCACAAGTGCCGTGCAGTCCCGCCCCCACTTGCTTCTCTTTGTGTGTAGTGTACGTACATTATCGAGACCGTTGTTCCCGCCCACCTCGATCCGGGGATCCCTGAGGTGTCTCACAAGTGCCGTGCAGTCCCGCCCCCACTTGCTTCTCTTTGTGTGTAGTGTACGTACATTATCGAGACCGTTGTTCCCGCCCACCTCGATCCGGGTCGACCTGAGGTGTCTCACAAGTGCCGTGCAGTCCCGCCCCCACTTGCTTCTCTTTGTGTGTAGTGTACGTACATTATCGAGACCGTTGTTCCCGCCCACCTCGATCCGGGAGCTCCTGAGGTGTCTCACAAGTGCCGTGCAGTCCCGCCCCCACTTGCTTCTCTTTGTGTGTAGTGTACGTACATTATCGAGACCGTTGTTCCCGCCCACCTCGATCCGGTCTAGACTGAGGTGTCTCACAAGTGCCGTGCAGTCCCGCCCCCACTTGCTTCTCTTTGTGTGTAGTGTACGTACATTATCGAGACCGTTGTTCCCGCCCACCTCGATCCGGGGATCCCTGAGGTGTCTCACAAGTGCCGTGCAGTCCCGCCCCCACTTGCTTCTCTTTGTGTGTAGTGTACGTACATTATCGAGACCGTTGTTCCCGCCCACCTCGATCCGGGTCGACCTGAGGTGTCTCACAAGTGCCGTGCAGTCCCGCCCCCACTTGCTTCTCTTTGTGTGTAGTGTACGTACATTATCGAGACCGTTGTTCCCGCCCACCTCGATCCGGGCATGCCTGCAGAAGCTTTTGTGGTTGGGACTTTAGCCAAGGGTATAAAAGACCACCGTCCCCGAATTACCTTTCCTCTTCTTTTCTCTCTCTCCTTGTCAACTCACACCCGAAATCGTTAAGCATTTCCTTCTGAGTATAAGAATCATTCAAAGGCGCGC |
| CrtB | ATGAACAACCCCTCTCTGCTGAACCACGCCGTGGAGACCATGGCCGTGGGCTCTAAGTCTTTCGCCACCGCCTCTAAGCTGTTCGACGCCAAGACCCGACGATCTGTGCTGATGCTGTACGCCTGGTGCCGACACTGCGACGACGTGATCGACGACCAGACCCTGGGCTTCCAGGCCCGACAGCCCGCCCTGCAGACCCCCGAGCAGCGACTGATGCAGCTGGAGATGAAGACCCGACAGGCCTACGCCGGCTCTCAGATGCACGAGCCCGCCTTCGCCGCCTTCCAGGAGGTGGCCATGGCCCACGACATCGCCCCCGCCTACGCCTTCGACCACCTGGAGGGCTTCGCCATGGACGTGCGAGAGGCCCAGTACTCTCAGCTGGACGACACCCTGCGATACTGCTACCACGTGGCCGGCGTGGTGGGCCTGATGATGGCCCAGATCATGGGCGTGCGAGACAACGCCACCCTGGACCGAGCCTGCGACCTGGGCCTGGCCTTCCAGCTGACCAACATCGCCCGAGACATCGTGGACGACGCCCACGCCGGCCGATGCTACCTGCCCGCCTCTTGGCTGGAGCACGAGGGCCTGAACAAGGAGAACTACGCCGCCCCCGAGAACCGACAGGCCCTGTCTCGAATCGCCCGACGACTGGTGCAGGAGGCCGAGCCCTACTACCTGTCTGCCACCGCCGGCCTGGCCGGCCTGCCCCTGCGATCTGCCTGGGCCATCGCCACCGCCAAGCAGGTGTACCGAAAGATCGGCGTGAAGGTGGAGCAGGCCGGCCAGCAGGCCTGGGACCAGCGACAGTCTACCACCACCCCCGAGAAGCTGACCCTGCTGCTGGCCGCCTCTGGCCAGGCCCTGACCTCTCGAATGCGAGCCCACCCCCCCCGACCCGCCCACCTGTGGCAGCGACCCCTGTAG |
| CrtE | ATGGCCATCTTCGCCGAGCGAGACTCTACCCTGATCTACTCTGACCCCCTGATGCTGCTGGCCATCATCGAGCAGCGACTGGACCGACTGCTGCCCGTGGAGTCTGAGCGAGACTGCGTGGGCCTGGCCATGCGAGAGGGCGCCCTGGCCCCCGGCAAGCGAATCCGACCCGTGCTGCTGATGCTGGCCGCCCACGACCTGGGCTACCGAGACGAGCTGTCTGGCCTGCTGGACTTCGCCTGCGCCGTGGAGATGGTGCACGCCGCCTCTCTGATCCTGGACGACATCCCCTGCATGGACGACGCCGAGCTGCGACGAGGCCGACCCACCATCCACCGACAGTTCGGCGAGCCCGTGGCCATCCTGGCCGCCGTGGCCCTGCTGTCTCGAGCCTTCGGCGTGATCGCCCTGGCCGACGGCATCTCTTCTCAGGCCAAGACCCAGGCCGTGGCCGAGCTGTCTCACTCTGTGGGCATCCAGGGCCTGGTGCAGGGCCAGTTCCTGGACCTGACCGAGGGCGGCCAGCCCCGATCTGCCGACGCCATCCAGCTGACCAACCACTTCAAGACCTCTGCCCTGTTCTCTGCCGCCATGCAGATGGCCGCCATCATCGCCGGCGCCCCCCTGGCCTCTCGAGAGAAGCTGCACCGATTCGCCCGAGACCTGGGCCAGGCCTTCCAGCTGCTGGACGACCTGACCGACGGCCAGTCTGACACCGGCAAGGACGCCCACCAGGACGTGGGCAAGTCTACCCTGGTGAACATGCTGGGCTCTAAGGCCGTGGAGAAGCGACTGCGAGACCACCTGCGACGAGCCGACCGACACCTGGCCTCTGCCTGCGACTCTGGCTACGCCACCCGACACTTCGTGCAGGCCTGGTTCGACAAGAAGCTGGCCATGGTGGGA |
| CrtI | ATGAAGCCCACCACCGTGATCGGCGCCGGCTTCGGCGGCCTGGCCCTGGCCATCCGACTGCAGGCCGCCGGCATCCCCGTGCTGCTGCTGGAGCAGCGAGACAAGCCCGGCGGCCGAGCCTACGTGTACGAGGACCAGGGCTTCACCTTCGACGCCGGCCCCACCGTGATCACCGACCCCTCTGCCATCGAGGAGCTGTTCGCCCTGGCCGGCAAGCAGCTGAAGGAGTACGTGGAGCTGCTGCCCGTGACCCCCTTCTACCGACTGTGCTGGGAGTCTGGCAAGGTGTTCAACTACGACAACGACCAGACCCGACTGGAGGCCCAGATCCAGCAGTTCAACCCCCGAGACGTGGAGGGCTACCGACAGTTCCTGGACTACTCTCGAGCCGTGTTCAAGGAGGGCTACCTGAAGCTGGGCACCGTGCCCTTCCTGTCTTTCCGAGACATGCTGCGAGCCGCCCCCCAGCTGGCCAAGCTGCAGGCCTGGCGATCTGTGTACTCTAAGGTGGCCTCTTACATCGAGGACGAGCACCTGCGACAGGCCTTCTCTTTCCACTCTCTGCTGGTGGGCGGCAACCCCTTCGCCACCTCTTCTATCTACACCCTGATCCACGCCCTGGAGCGAGAGTGGGGCGTGTGGTTCCCCCGAGGCGGCACCGGCGCCCTGGTGCAGGGCATGATCAAGCTGTTCCAGGACCTGGGCGGCGAGGTGGTGCTGAACGCCCGAGTGTCTCACATGGAGACCACCGGCAACAAGATCGAGGCCGTGCACCTGGAGGACGGCCGACGATTCCTGACCCAGGCCGTGGCCTCTAACGCCGACGTGGTGCACACCTACCGAGACCTGCTGTCTCAGCACCCCGCCGCCGTGAAGCAGTCTAACAAGCTGCAGACCAAGCGAATGTCTAACTCTCTGTTCGTGCTGTACTTCGGCCTGAACCACCACCACGACCAGCTGGCCCACCACACCGTGTGCTTCGGCCCCCGATACCGAGAGCTGATCGACGAGATCTTCAACCACGACGGCCTGGCCGAGGACTTCTCTCTGTACCTGCACGCCCCCTGCGTGACCGACTCTTCTCTGGCCCCCGAGGGCTGCGGCTCTTACTACGTGCTGGCCCCCGTGCCCCACCTGGGCACCGCCAACCTGGACTGGACCGTGGAGGGCCCCAAGCTGCGAGACCGAATCTTCGAGTACCTGGAGCAGCACTACATGCCCGGCCTGCGATCTCAGCTGGTGACCCACCAGATGTTCACCCCCTTCGACTTCCGAGACCAGCTGAACGCCTACCAGGGCTCTGCCTTCTCTGTGGAGCCCGTGCTGACCCAGTCTGCCTGGTTCCGACCCCACAACCGAGACAAGACCATCACCAACCTGTACCTGGTGGGCGCCGGCACCCACCCCGGCGCCGGCATCCCCGGCGTGATCGGCTCTGCCAAGGCCACCGCCGGCCTGATGCTGGAGGACCTGATCTAG |

**Table S3.** Sequencing of disrupted genes. Underlined sequences show PAM sequences, and red bases indicate variations from the wildtype sequence that result in frameshift mutations.

| HEBI | TATCTCTACCTCTTCCGCTGCTGGTCTTTACGGAAACTTCG |
| --- | --- |
| HEBI ΔMFE1 | TATCTCTACCTCTTCCGCTG-TGGTCTTTACGGAAACTTCG |
|  |  |
|  |  |
| HEBI | CTTGCTCTGAACGACTCTAA-ACAGGGTGGCGACAGCAAGC |
| HEBI ΔPAH1 | CTTGCTCTGAACGACTCTAAAACAGGGTGGCGACAGCAAGC |
|  |  |
|  |  |
| HEBI | CCTGCTGTTCGAGGTCGCCA-CCGAGGTCGCCAACCGGGTC |
| HEBI ΔGSY1 | CCTGCTGTTCGAGGTCGCCAACCGAGGTCGCCAACCGGGTC |


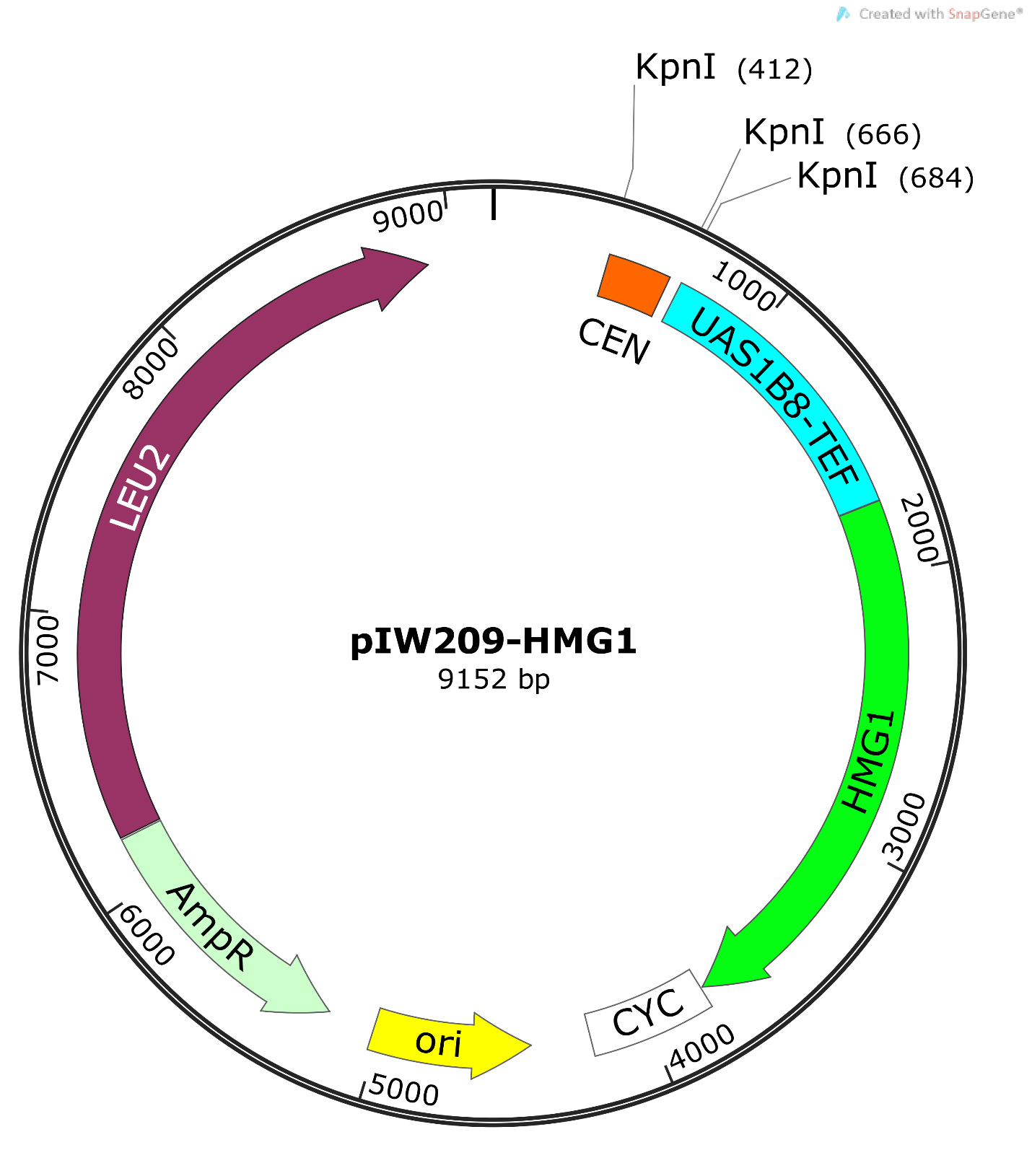


**Figure S1**. Plasmid map of pIW209-HMG1 gene used for overexpression during linear transformation. Digestion with KpnI used for linearization.


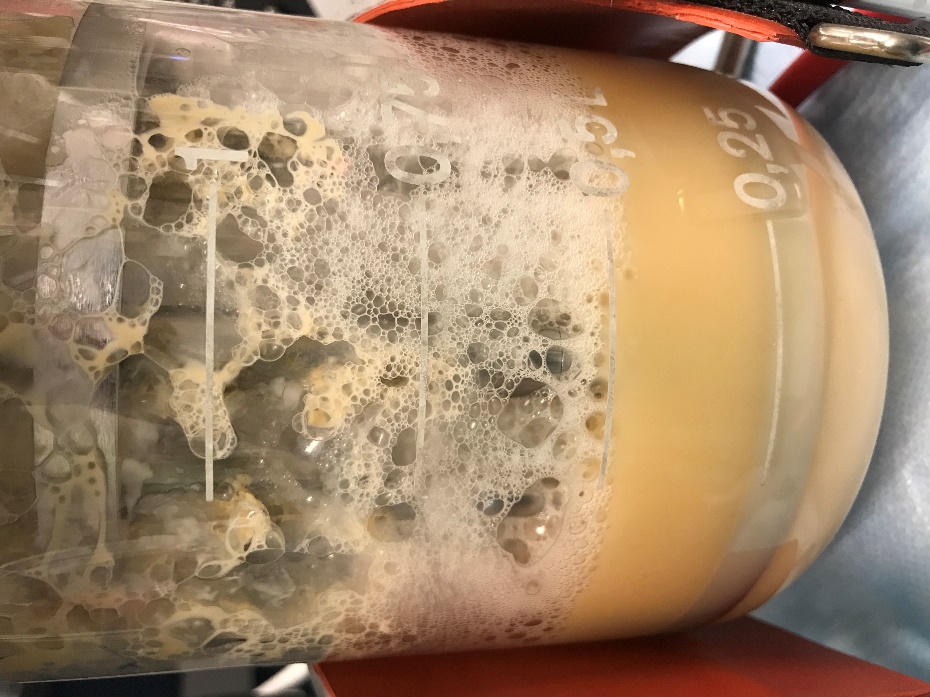

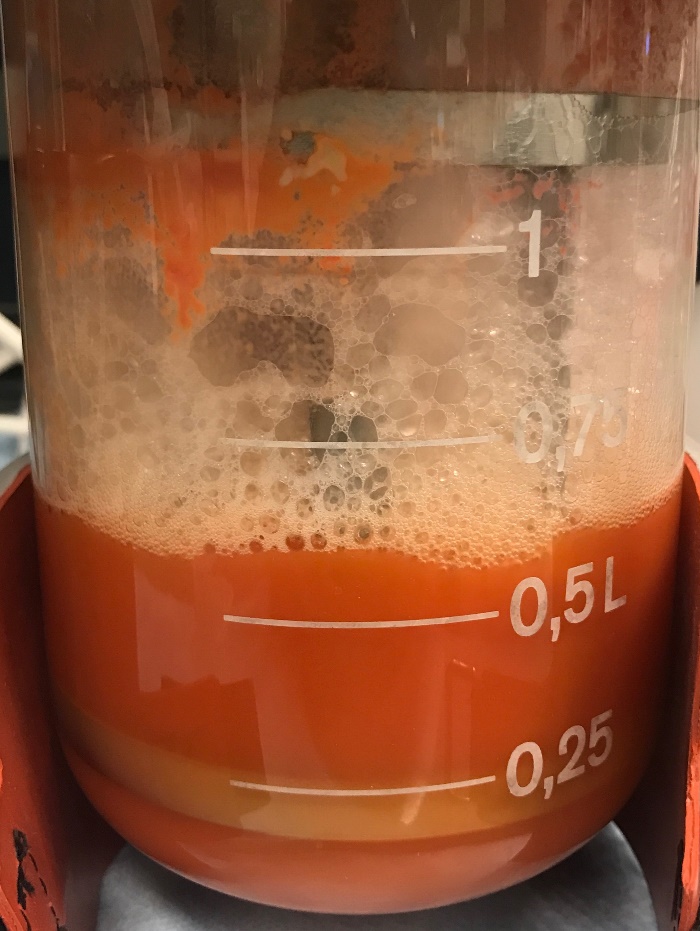


**Figure S2.** Image of the bioreactor at day 1 (left) and day 10 (right) showing lycopene accumulation.


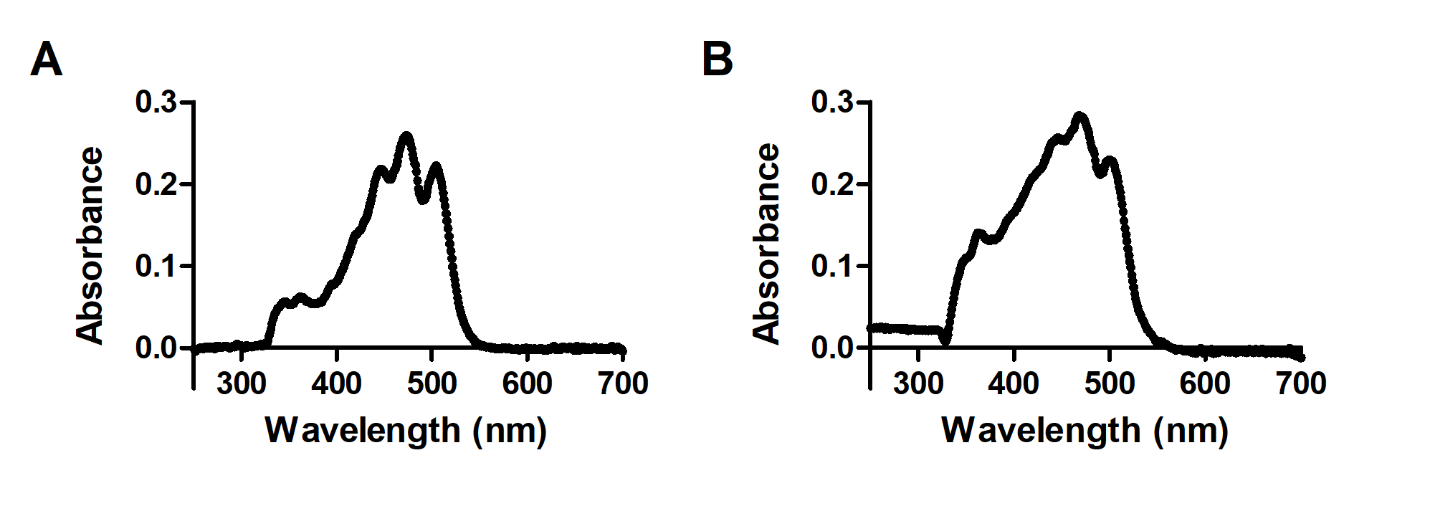


**Figure S3.** Absorbance spectrum of lycopene. (A) Absorption spectrum of purchased lycopene in acetone. (B) Absorption spectrum of lycopene produced by and extracted from *Y. lipolytica* in acetone.
